# Observer-Based Optimal Control of a Quadplane with Active Wind Disturbance and Actuator Fault Rejection

**DOI:** 10.3390/s23041954

**Published:** 2023-02-09

**Authors:** Zaidan Zyadat, Nadjim Horri, Mauro Innocente, Thomas Statheros

**Affiliations:** 1 Autonomous Vehicles & Artificial Intelligence Laboratory (AVAILAB), Centre for Future Transport and Cities, Faculty of Engineering, Environment and Computing, Coventry University, Coventry CV1 5FB, UK; 2 Centre for Future Transport and Cities, Faculty of Engineering, Environment and Computing, Coventry University, Coventry CV1 5FB, UK; 3 School of Future Transport Engineering, Faculty of Engineering, Environment and Computing, Coventry University, Coventry CV1 5FB, UK

**Keywords:** UAV, quadplane, observer, actuator fault, wind disturbance, active disturbance rejection, optimal control, LQR, transitioning

## Abstract

Hybrid aircraft configurations with combined cruise and vertical flight capabilities are increasingly being considered for unmanned aircraft and urban air mobility missions. To ensure the safety and autonomy of such missions, control challenges including fault tolerance and windy conditions must be addressed. This paper presents an observer-based optimal control approach for the active combined fault and wind disturbance rejection, with application to a quadplane unmanned aerial vehicle. The quadplane model is linearised for the longitudinal plane, vertical takeoff and landing and transition modes. Wind gusts are modelled using a Dryden turbulence model. An unknown input observer is first developed for the estimation of wind disturbance by defining an auxiliary variable that emulates body referenced accelerations. The approach is then extended to simultaneous rejection of intermittent elevator faults and wind disturbance velocities. Estimation error is mathematically proven to converge to zero, assuming a piecewise constant disturbance. A numerical simulation analysis demonstrates that for a typical quadplane flight profile at 100 m altitude, the observer-based wind gust and fault correction significantly enhances trajectory tracking accuracy compared to a linear quadratic regulator and to a H-infinity controller, which are both taken, without loss of generality, as benchmark controllers to be enhanced. This is done by adding wind and fault compensation terms to the controller with admissible control effort. The proposed observer is also shown to enhance accuracy and observer-based rejection of disturbances and faults compared to three alternative observers, based on output error integration, acceleration feedback and a sliding mode observer, respectively. The proposed approach is particularly efficient for the active rejection of actuator faults under windy conditions.

## 1. Introduction

There is a growing interest in the development of electric vertical takeoff and landing (eVTOL) aircrafts because of their potential for urban air mobility and a wide range of unmanned aerial vehicle (UAV) applications. The global eVTOL market is indeed currently forecasted to more than triple in size by 2030 [1]. There is also growth in the use of hybrid aircraft configurations in the UAV market. The quadplane concept with independent thrust is considered in this paper because it is emerging as a popular UAV configuration due to its relative simplicity and ability to switch between cruising like a plane and VTOL flight as a quadcopter, herein referred to as plane and quad modes, respectively.

The emerging eVTOL and hybrid UAV concepts require the development of autonomous but safe control systems. In the federal aviation administration (FAA) legislation on UAVs, autonomy includes the ability to handle faults. The handling of winds is also an important safety consideration and a known weakness of hybrid aircraft configurations such as quadplanes [2]. Unknown input observers have been used for real-time estimations of exogenous inputs, such as rotor efficiency loss faults in hexacopters [3], and to estimate multiple quadcopter rotor faults simultaneously by using nonlinear observers [4]. Sliding mode observers were also combined with incremental sliding mode control to enable the adaptive fault tolerant control of quadplanes with improved robustness to one rotor loss, compared to conventional linear quadratic regulator (LQR) based trajectory tracking [5,6]. Linear unknown input observers have also been used for the detection of icing [7], and for the diagnosis of fixed-wing UAV icing and actuator faults [8]. Kalman filter (KF) based approaches were also used for the estimation of winds in UAV where a multiplicative KF is used [9], and where a two-stage KF is used [10]. In the latter study, wind estimation is obtained by an extended Kalman filter (EKF) and sensor fault estimation is obtained at a second stage using a robust KF. Observers remain the preferred approach when convergence to the fault or disturbance has more priority than accurate state estimation under sensor and process noise. In [11], sliding mode control was combined with a perturbation observer to reduce chattering with robustness to trigonometric perturbations without explicitly modelling wind gusts. Fault and disturbance rejection have rarely been jointly considered for small quadcopters and conventional aircraft, but not for hybrid aircraft such as quadplanes. In [12], a combined fault and disturbance observer was used to enable fault tolerant control of a quadrotor using an adaptive fuzzy state observer with terminal SMC. In [13], an extended state observer (ESO) was used to estimate an augmented state vector with state and fault components, using an ESO-LQR loop to control a tilt-rotor quadplane in hover. In [14], linear control was combined with an ESO for quadplane path following and to compensate disturbances that did not follow a formal wind model.

Transitioning between the quad (VTOL) and plane flight modes is another control challenge in hybrid aircraft configurations. In [15], a mode switching hybrid system model was used, including linear plane, quad and transition modes: the transition controllers used prescribed exponential decay and convergence functions to progressively decrease cruise speeds and increase quad inputs during a transition to quad mode, and inverted this logic to transition to plane mode. In [16,17], a similar mode-switching control approach was successfully flight tested for quadplane control using a weighted sum of quad and plane actuator inputs during transitioning, but controllers did not use a detailed hybrid dynamical model. In [16], the weights of the plane and quad commands during transitioning were based on a transition time fraction. In [17], the weights were dependent on a cruise speed fraction. A similar weighted sum transitioning approach was described in [18] for hybrid UAV, flight tested on a tailsitter in [19], a quadplane in [20] and on a tiltrotor fixed-wing in [21]. The controllers in [15,16,17,18,19,20] implemented transitioning without wind rejection or fault recovery. In [22], a gain scheduled LQR approach was used for the weighting of plane and quad commands during the transitioning stage of an eVTOL tiltwing UAV and a pseudoinversion process is used with a perturbation observer for feedforward wind gust compensation. A simplified discrete wind gust model was used based FAA documentation. In practice, it is important that the longitudinal and vertical speed flight envelopes of the transitioning modes overlap with those of plane and flight modes [23]. In [24], different transitioning strategies were used for different flight profiles, including conventional vertical or bird-like takeoff. Challenges in transitioning partly explain why quadplane flight tests in some papers are focused on demonstrating the ability to hover, as in [25]. In [26], machine learning was applied to the quadplane transitioning problem using a neural network with a Lyapunov based update law and a deep deterministic policy gradient method to solve the Bellman equation.

The control of quadplanes and other hybrid UAV with disturbance rejection or fault tolerance is often based on robust variants of SMC. In [27], supertwisting SMC was used to mitigate the impact of disturbances and model uncertainty on quadplane trajectory following. In [28], integral sliding mode control was used to ensure fault tolerant control in an overactuated UAV, with a hardware-in-the-loop (HIL) validation. Other approaches use observer-based controllers as previously described in [5,6,11,14]. In [29], an adaptive multiple model approach was also shown to achieve efficient control of a quadplane using different models for the plane, quad and transition modes, with real-time updates of the trim conditions during transitioning. In [30], an ESO based disturbance rejection altitude controller was applied to a small Nano Talon UAV with weighted transitioning. In [31], a similar approach was used to reject disturbances affecting the pitch rate in an eVTOL.

For the modelling of wind gusts effects in different types of aircraft, the Von Karman [2,32,33,34] and Dryden [34,35,36,37] models are the most popular power spectral density-based models based on isotropic turbulence theory, with empirically derived coefficients [34]. The Von Karman model was used for studies focused on the electric power consumption of the VTOL mode of quadplanes under winds in [33]. Despite the Von Karman model being slightly more accurate at predicting higher wind frequencies, the Dryden wind model is adopted here, as it simplifies the conversion to time domain disturbance.

In this paper, a typical quadplane VTOL flight profile is considered, with a gradual transitioning between the plane and quad modes. Given the winds handling weaknesses of quadplanes and the importance of fault tolerance for safety and legislation considerations, an unknown input observer approach is developed to ensure accurate quadplane path following under both winds and actuator faults. The quadplane model under consideration is based on the Aerosonde twin boom design, which is popular in the research literature (see [28,38]) and employed in industry by companies, such as Textron Systems Corporation. Axial thrust is independently provided by a pusher propeller.

The main contributions of the paper are:

- An unknown input observer with a theoretical convergence proof is applied to real-time simultaneous wind gust velocities and actuator fault estimation. Compared to three classical observers used for comparison, this observer is shown to significantly improve estimation accuracy. The three alternative observers are inspired from [30], where output error integration is used, [39], where the observer uses acceleration measurements as indirect disturbance observations and [40], where a sliding mode observer (SMO) was used. The proposed observer uses an auxiliary variable to avoid acceleration measurements and is simple to tune for exponential convergence.
- An observer-based control approach with wind gusts and actuator fault rejection under the plane, quad and transitioning modes, which significantly enhances path following accuracy. This approach combines a linear quadratic regulator (LQR) with a H¥ controller with exact wind and fault compensation based on a pseudoinversion process. LQR and H¥ control are used without loss of generality as benchmark controllers to be enhanced. The control architecture is simple to implement by separating state and perturbation estimation.
- A linearised quadplane model with disturbance and fault inputs, with analytical trim conditions for the plane and quad modes and numerical trimming for the transition modes. A speed dependent weighted transitioning logic is used between the quad and plane modes.

The paper is organised as follows. In Section 2, a nonlinear dynamical quadplane model is developed and linearised for the plane, quad and transition modes and a Dryden wind gust model is presented. Section 3 introduces the Luenberger state observer and the unknown input observer, which is first applied to wind gusts estimation before being extended to simultaneous wind and fault estimation, with a mathematical proof of error decay. The LQR reference following controller with observer-based combined wind gusts and fault rejection is presented in Section 4. A numerical simulation analysis of observer estimation accuracy and of control performance using the proposed observer-based quadplane control approach is presented in Section 5. This includes a comparison against the three abovementioned observers; the observer-based correction is added to LQR then H∞ trajectory tracking control. Section 6 concludes the paper.

## 2. Quadplane Dynamic and Kinematic Models

In this section, we derive the quadplane nonlinear dynamics equations in vector and components form before focusing on the longitudinal dynamics and model linearisation of the quadplane model for all flight modes.

### 2.1. Nonlinear Aircraft Dynamics

The vector form nonlinear translational dynamics of the quadplane can be described by Newton’s second law as follows:

(1) $$F_{b}=m({\dot{V}}_{b}+\omega_{b}\times V_{b})$$

where $V_{b}$ represents the velocity vector of the aircraft, expressed in body coordinates, $\omega_{b}$ represents the angular velocity vector of the body frame with respect to the inertial frame, also expressed in body coordinates and $F_{b}$ represents the sum of all external forces in the body frame.

Newton’s second law for the rotational motion of the quadplane is given by:

(2) $$m_{b}=J{\dot{\omega }}_{b}+\omega_{b}\times J\omega_{b}$$

where $m_{b}$ represents the sum of all external moments, expressed in the UAV body frame and **J** is the moment of inertia matrix.

**Figure 1:**
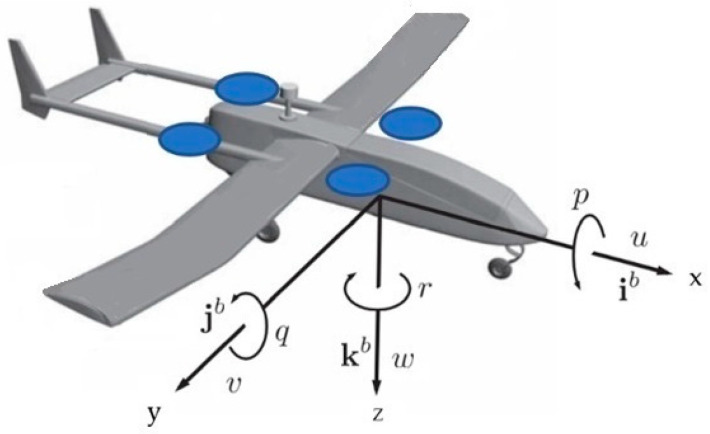
Aircraft body axes convention. The figure was taken and adapted from [5].

In Equations (1) and (2), the components of $\omega_{b}$ along the x, y and z body axes of the aircraft represent the roll rate p, pitch rate q and yaw rate r, respectively, as shown in Figure 1. The components of the velocity vector $V_{b}$ along the x, y and z body axes are the longitudinal speed u, lateral speed v and vertical descent speed w.

### 2.2. Longitudinal Dynamics and Kinematics Equations

In this paper, the focus is on the longitudinal dynamics of the quadplane, as in many references. Equations (1) and (2) were valid for the full six degrees of freedom (6DoF) motion but are also valid for the longitudinal dynamics case by redefining $F_{b}={\left[F_{X},F_{Z}\right]}^{T}$, $m_{b}=M$, $\omega_{b}=q$ and $V_{b}={\left[u,w\right]}^{T}$ where $F_{X},F_{Z}$ are the resultant forces along the x and z body axes and *M* is the total pitching moment acting on the quadplane. For longitudinal manoeuvres satisfying r=p=v=0, and without lateral control inputs, the longitudinal dynamics are indeed decoupled from the lateral ones. The differential equations for longitudinal dynamics of the quadplane are given by [29,38]:

(3) $$\begin{array}{c} \dot{u}=-qw+\frac{F_{X}}{m}\\ \dot{w}=qu+\frac{F_{Z}}{m}\\ \dot{q}=\frac{M}{J_{y}}\\ \dot{\theta }=q \end{array}$$

where $J_{y}$ is the moment of inertia about the pitch axis and *m* is aircraft mass.

The kinematic equations for the aircraft north and down positions $p_{n}$ and $p_{d}$ with respect to the local x and z Earth fixed axes, respectively, can then easily be obtained by considering the pitch rotation θ from the inertial to the body frame:

(4) $$\left[\begin{array}{c} {\dot{p}}_{n}\\ {\dot{p}}_{d} \end{array}\right]=\left[\begin{array}{cc} \cos\left(\theta \right) & \sin(\theta )\\ -\sin\left(\theta \right) & \cos\left(\theta \right) \end{array}\right]\left[\begin{array}{c} u\\ w \end{array}\right]$$

### 2.3. Forces and Moments Equations

**Figure 2:**
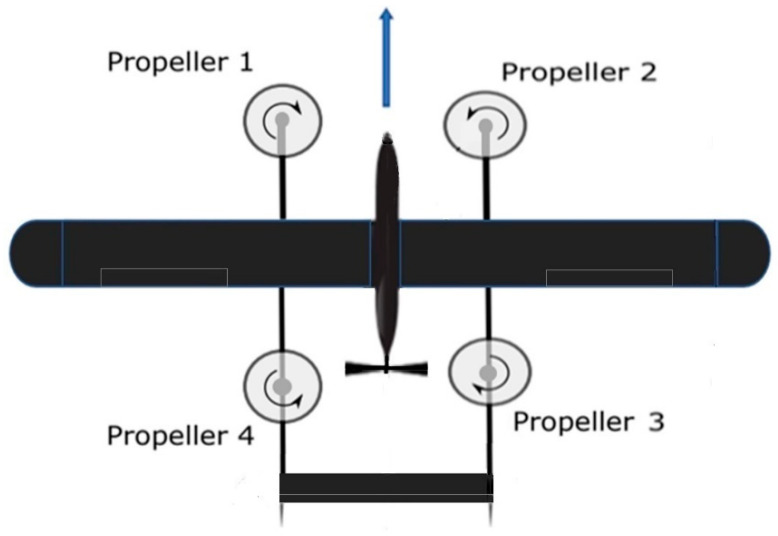
Quadplane rotors configuration.

The quadplane configuration is depicted in Figure 2. The configuration consists of four rotors in an X-configuration and installed on a fixed-wing plane. The quadplane is a hybrid aircraft that employs separate propulsion systems for the VTOL and plane modes.

The resultant force vector acting on the quadplane is given by

(5) $$F_{b}=F_{g}+F_{A}+F_{\mathrm{prop}}+F_{d}$$

where $F_{A}$ and $F_{\mathrm{prop}}$ represent the aerodynamic and propulsion forces, respectively, and $F_{d}={\left[F_{Xd},F_{Zd}\right]}^{T}$ denotes the disturbance force due to wind gusts and faults in the (x,z) plane. In the following, the quadrotor forces and moments will be considered to be part of the aerodynamic force, which is given by $F_{A}=F_{p}+F_{\mathrm{Zquad}}$, where $F_{p}={\left[F_{Xp},F_{Zp}\right]}^{T}$ represents the plane commands and all airframe related longitudinal aerodynamic forces and $F_{\mathrm{Zquad}}$ represents the lift force of the quad inputs, which only acts along the z body axis. In the (x,z) plane, the scalar moment about the pitch axis is the sum of the moment due to plane commands $M_{p}$ and one due to quad commands $M_{quad}$:

(6) $$m_{b}=M_{p}+M_{quad}+M_{d}$$

where $M_{d}$ is a pitching moment disturbance, typically due to the wind disturbance and potential additive actuator faults. The focus of this report will be on the longitudinal dynamics with forces and translations in the (x,z) plane and moments and rotations about the y axis (pitch axis) of the body frame.

#### 2.3.1. Gravitational Forces

Assuming longitudinal motion, the gravitational force acting on the UAV is given by

(7) $$F_{g}=\left(\begin{array}{c} -mg\;\sin\left(\theta \right)\\ mg\;\cos\left(\theta \right) \end{array}\right)$$

where g is the Earth’s gravitational acceleration.

#### 2.3.2. Plane Commands Related Aerodynamic Forces and Moments

The forces and moments acting in the (x,z) plane are the lift and drag forces in addition to the pitching moment and are considered as parts of the plane model. The quad commands constitute additional inputs to this model. The aerodynamic forces and moments are influenced by the elevator deflection η, angle of attack α and pitch rate q. The throttle or axial propulsion force is considered separately, as previously described.

Due to plane dynamics based on a first order Taylor series expansion of the effects of dynamical variables and plane inputs, the lift, drag and pitching moments are

(8) $$L_{p}=\frac{1}{2}\rho V_{a}{}^{2}S\left[C_{L_{0}}+C_{L_{\alpha }}\alpha +C_{L_{q}}\frac{c}{2V_{a}}q+C_{L_{\eta }}\eta \right]$$

(9) $$D_{p}=\frac{1}{2}\rho V_{a}{}^{2}S\left[C_{D_{0}}+C_{D_{\alpha }}\alpha +C_{D_{q}}\frac{c}{2V_{a}}q+C_{D_{\eta }}\eta \right]$$

(10) $$M_{p}=\frac{1}{2}\rho V_{a}{}^{2}S\left[C_{m_{0}}+C_{m_{\alpha }}\alpha +C_{m_{q}}\frac{c}{2V_{a}}q+C_{m_{\eta }}\eta \right]$$

where the *p* index denotes the plane mode, ρ is the atmospheric density, $V_{a}$ is resultant speed, *S* is the reference area and all other parameters are lift coefficient, drag coefficient and pitching moment coefficient stability derivatives representing the effect of variables in their subscript. For example, $C_{M_{q}}$ represents the effect of pitch rate on the pitching moment, also known as pitch damping coefficient.

The x and z axis components of the force acting on the UAV under plane commands and their associated dynamics can then be related to the plane lift and drag forces:

(11) $$\left(\begin{array}{c} F_{Xp}\\ F_{Zp} \end{array}\right)=\left(\begin{array}{cc} \cos\left(\alpha \right) & -\sin\left(\alpha \right)\\ \sin\left(\alpha \right) & \cos\left(\alpha \right) \end{array}\right)\left(\begin{array}{c} -D_{p}\\ -L_{p} \end{array}\right)$$

#### 2.3.3. Quad Forces and Moments

A quadrotor cross configuration is assumed where the four rotors of the quadplane are labelled from 1 to 4, as shown in Figure 2.

The quad mode controls a vector, $u_{q}={\left[F_{Zq},M_{q}\right]}^{T}$, where $F_{Zq}$ represents the sum of the lift forces of all four rotors in the body frame, which is proportional to the sum of the squared rotor speeds:

(12) $$F_{Zquad}=-b\left(\Omega_{1}{}^{2}+\Omega_{2}{}^{2}+\Omega_{3}{}^{2}+\Omega_{4}{}^{2}\right)$$

where *b* is the thrust coefficient of all four rotors and $\Omega_{i},i=1,4$ are the rotor speeds. The minus sign is based on the convention that the z axis points downwards.

The quad commanded pitching moment $M_{quad}$ is obtained as the difference between two pairs of rotors forces:

(13) $$M_{quad}=bl\left(\Omega_{1}{}^{2}+\Omega_{2}{}^{2}-\Omega_{3}{}^{2}-\Omega_{4}{}^{2}\right)$$

where l is the arm length of the rotors. Given that the focus is on longitudinal motion, it can be assumed that $\Omega_{1}=$ $\Omega_{2}$ and $\Omega_{3}=$ $\Omega_{4}$.

#### 2.3.4. Throttle Force

The throttle input τ is used for axial propulsion and is related to propulsion force acting on the x axis by the following Equation [38]:

(14) $$F_{\mathrm{prop}}=\frac{1}{2}S_{prop}C_{prop}\left(\begin{array}{c} {\left(K_{motor}\tau \right)}^{2}-V_{a}{}^{2}\\ 0\\ 0 \end{array}\right)$$

where $S_{prop}$ is the area swept by the axial propeller, $C_{prop}$ is a propulsion system coefficient, $K_{motor}$ is the proportionality constant between the air exhaust velocity and the throttle input and $V_{a}$ is the airspeed. The first component of $F_{\mathrm{prop}}$ is denoted $f_{prop}$. Note that the square of the throttle in (14) is linearised.

#### 2.3.5. Total Longitudinal Forces and Moments

The longitudinal airframe aerodynamic forces and the pitching moment in the body frame of the quadplane are respectively given by

(15) $$F_{Xp}=\frac{1}{2}\rho V_{a}{}^{2}S\left(C_{X}\left(\alpha \right)+C_{X_{q}}\left(\alpha \right)\frac{c}{2V_{a}}q+C_{X_{\eta }}\left(\alpha \right)\eta \right)$$

(16) $$F_{Zp}=\frac{1}{2}\rho V_{a}{}^{2}S\left(C_{Z}\left(\alpha \right)+C_{Z_{q}}\left(\alpha \right)\frac{c}{2V_{a}}q+C_{Z_{\eta }}\left(\alpha \right)\eta \right)$$

(17) $$M_{p}=\frac{1}{2}\rho V_{a}{}^{2}Sc\left(C_{m_{0}}+C_{m_{q}}\alpha +C_{m_{q}}\frac{c}{2V_{a}}q+C_{m_{\eta }}\eta \right)$$

After adding the quad forces and moments and axial propulsion and gravity forces, we have the following expressions for the components of the total force on the x and z body axes:

(18) $$F_{X}=F_{Xp}-mg\sin\left(\theta \right)-f_{prop}+F_{Xd}$$

(19) $$F_{Z}=F_{Zp}+F_{Zquad}+mg\cos\left(\theta \right)+F_{Zd}$$

where $F_{Xd},F_{Zd}$ are the x and z body-referenced components of the force due to wind disturbance and additive actuator faults.

In Equation (19), it is assumed that the drag of the quad structure is included in $F_{Xp}$, which is treated as part of the plane structure.

### 2.4. Quadplane Model Linearisation

The nonlinear quadplane dynamics can be written compactly as

(20) $$\dot{x}=f\left(x,u,d_{g},f_{a}\right)$$

where $x={\left[u,w,q,\theta ,p_{d}\right]}^{T}$ is the state vector, $u={\left[\eta ,\tau ,F_{ZQuad},M_{Quad}\right]}^{T}$ is the control inputs vector. The external wind disturbance vector $d_{g}={\left[u_{g},w_{g},q_{g}\right]}^{T}$ has two wind gust velocity components $u_{g},w_{g}$ along the x and z body axes, respectively, and a pitch rate component $q_{g}$. The components of vector $f_{a}={\left[f_{\delta },f_{\tau }\right]}^{T}$ represent additive faults on the elevator and throttle inputs. Model linearisation is obtained by deriving the nonlinear dynamics function *f* at the trim point ($x^{*},u^{*}$), where $x^{*}$ and $u^{*}$ denote the trim conditions for the states and control inputs, respectively, with zero trim conditions for all elements of $d_{g}$ and $f_{a}$. The value of the trim points $x^{*}={\left[U^{*},W^{*},q^{*},\theta^{*},p_{d}{}^{*}\right]}^{T}$ and $u^{*}={\left[\eta^{*},\tau^{*},F_{Zquad}^{*},M_{quad}^{*}\right]}^{T}$ can be obtained from

(21) $${\dot{x}}^{*}=f\left(x^{*},u^{*}\right)=0$$

By defining $\dot{\bar{x}}=\dot{x}-\dot{x^{*}}$ is a variation with respect to the trim condition, we have

(22) $$\dot{\bar{x}}=f\left(x^{*}+\bar{x},u^{*}+\bar{u}\right)-f\left(x^{*},u^{*}\right)$$

Using Taylor’s expansion about the trim point up to the first order, we have

(23) $$\dot{\bar{x}}=\frac{\partial f\left(x^{*},u^{*}\right)}{\partial x}\bar{x}+\frac{\partial f\left(x^{*},u^{*}\right)}{\partial u}\bar{u}$$

The linearised system can therefore be obtained by taking the derivatives of the function *f* with respect to x and u at the trim point.

The linearisation assumptions are that all lateral states are assumed to be zero, the angle of attack is a function of the vertical velocity: *α* $=\tan^{-1}\left(\frac{w}{U^{*}}\right)$ and $U^{*}$ is the trimmed axial velocity, with small speed and pitch variations with respect to trim. The norm of the velocity vector with respect to the trim condition $V_{a}$ satisfies $V_{a}{}^{2}=u^{2}+w^{2}$.

In all modes, the linearised state-space model is similar to the one in [15] with the addition of disturbance and fault channels similarly to [40] as follows:

(24) $$\dot{x}=A_{\mathrm{mode}}\;x+B_{\mathrm{mode}}\left(u_{\mathrm{mode}}+f_{\mathrm{mode}}\right)+B_{\mathrm{gmode}}d_{g}$$

where the state matrix $A_{\mathrm{mode}}$ is denoted $A_{p},A_{q}$ and $A_{\mathrm{tr}}$, and $B_{\mathrm{mode}}$ is also denoted $B_{p},B_{q}$ and $B_{\mathrm{tr}}$ in the plane, quad and transition modes, respectively. The same indexation logic is used for $B_{\mathrm{gmode}}$ and the vector $f_{\mathrm{mode}}$ represents the potentially faulty actuators in the mode. At each mode, the state matrix is obtained by partial differentiation of the function *f* with respect to the state vector x at a trim condition for the mode.


(25) $$A_{\mathrm{mode}}={\left[\frac{\partial f_{long}}{\partial x_{long}}\right]}_{x=x^{*}\left(\mathrm{for}\;\mathrm{the}\;\mathrm{mode}\right)}={\left[\begin{array}{ccccc} \frac{\partial \dot{u}}{\partial u} & \frac{\partial \dot{u}}{\partial w} & \frac{\partial \dot{u}}{\partial q} & \frac{\partial \dot{u}}{\partial \theta } & \frac{\partial \dot{u}}{\partial p_{d}}\\ \frac{\partial \dot{w}}{\partial u} & \frac{\partial \dot{w}}{\partial w} & \frac{\partial \dot{w}}{\partial q} & \frac{\partial \dot{w}}{\partial \theta } & \frac{\partial \dot{w}}{\partial p_{d}}\\ \frac{\partial \dot{q}}{\partial u} & \frac{\partial \dot{q}}{\partial w} & \frac{\partial \dot{q}}{\partial q} & \frac{\partial \dot{q}}{\partial \theta } & \frac{\partial \dot{q}}{\partial p_{d}}\\ \frac{\partial \dot{\theta }}{\partial u} & \frac{\partial \dot{\theta }}{\partial w} & \frac{\partial \dot{\theta }}{\partial q} & \frac{\partial \dot{\theta }}{\partial \theta } & \frac{\partial \dot{\theta }}{\partial p_{d}}\\ \frac{\partial \dot{p}{}_{d}}{\partial u} & \frac{\partial \dot{p}{}_{d}}{\partial w} & \frac{\partial \dot{p}{}_{d}}{\partial q} & \frac{\partial \dot{p}{}_{d}}{\partial \theta } & \frac{\partial \dot{p}{}_{d}}{\partial \theta } \end{array}\right]}_{x=x^{*}\left(\mathrm{for}\;\mathrm{the}\;\mathrm{mode}\right)}$$

Likewise, the control matrix is obtained by partial differentiation with respect to control inputs vector at the trim condition for the mode.

(26) $$\begin{array}{c} B_{\mathrm{mode}}={\left[\frac{\partial f_{long}}{\partial u_{long}}\right]}_{u=u^{*}\left(\mathrm{for}\;\mathrm{the}\;\mathrm{mode}\right)}={\left[\begin{array}{c} \begin{array}{cc} \frac{\partial \dot{u}}{\partial \eta } & \frac{\partial \dot{u}}{\partial \tau }\\ \frac{\partial \dot{w}}{\partial \eta } & \frac{\partial \dot{w}}{\partial \tau }\\ \frac{\partial \dot{q}}{\partial \eta } & \frac{\partial \dot{q}}{\partial \tau }\\ \frac{\partial \dot{\theta }}{\partial \eta } & \frac{\partial \dot{\theta }}{\partial \tau } \end{array}\\ \begin{array}{cc} \frac{\partial {\dot{p}}_{d}}{\partial \eta } & \frac{\partial {\dot{p}}_{d}}{\partial \tau } \end{array} \end{array}\right]}_{u=u^{*}\left(\mathrm{for}\;\mathrm{the}\;\mathrm{mode}\right)} \end{array}$$

The matrix $B_{\mathrm{gmode}}$ is obtained similarly by differentiation with respect to $d_{g}$ (see [38]). This paper is focused on the case of additive faults on the plane inputs only, but under windy conditions. We therefore have $f_{\mathrm{mode}}=f_{a}$ during the plane and transition modes when plane commands are active. In the quad mode, plane commands are turned off, so it is realistic to assume that no plane input faults are present, although our algorithms can easily be adapted to handle biases due to plane commands in any mode.

#### 2.4.1. Plane Model

The plane mode is described by a conventional airplane model (see [38] for the aircraft model and [40] for the addition of disturbance inputs channels) with additional terms for the additive fault and wind disturbance. The state-space model is given by the following:

(27) $$\dot{x}=A_{p}x+B_{p}\left(u_{p}+f_{a}\right)+B_{\mathrm{gp}}d_{g}$$

The state matrix $A_{p}$ of the plane mode is obtained for any trim condition on $U^{*},$ $W^{*},\theta^{*}$ as shown in Equation (28):

(28) $$A_{p}=\left[\begin{array}{ccccc} X_{u} & X_{w} & X_{q} & -g\cos(\theta^{*}) & 0\\ Z_{u} & Z_{w} & Z_{q} & -g\sin(\theta^{*}) & 0\\ M_{u} & M_{w} & M_{q} & 0 & 0\\ 0 & 0 & 1 & 0 & 0\\ \sin(\theta^{*}) & -\cos(\theta^{*}) & 0 & U^{*}\cos(\theta^{*})+W^{*}\sin(\theta^{*}) & 0 \end{array}\right]$$

The control and wind disturbance input matrices are given by [38], respectively:

(29) $$B_{p}=\left[\begin{array}{cc} X_{\eta } & X_{\tau }\\ Z_{\eta } & Z_{\tau }\\ M_{\eta } & M_{\tau }\\ 0 & 0\\ 0 & 0 \end{array}\right]$$

(30) $$B_{\mathrm{gp}}=\left[\begin{array}{ccc} X_{u} & X_{w} & X_{q}\\ Z_{u} & Z_{w} & Z_{q}\\ M_{u} & M_{w} & M_{q}\\ 0 & 0 & 0\\ 0 & 0 & 0 \end{array}\right]$$

and the plane mode control vector is given by $u_{p}={\left[\eta ,\tau \right]}^{T}$. In practice, a cruise trim condition is generally used for the plane mode of a quadplane, and in this paper $U^{*}=20$ m/s and $W^{*}=q^{*}=\theta^{*}$ = 0. The trim condition on altitude has less influence on the stability derivatives because sea level density can be assumed at the relatively low altitudes considered here. The terms $X_{u},X_{w},X_{q},$ $Z_{u},$ $Z_{w},$ $Z_{q},$ $M_{u},$ $M_{w}$ and $M_{q}$ are the longitudinal stability derivatives and $X_{\eta },Z_{\eta }$, $M_{\eta },X_{\tau },Z_{\eta }$ and $M_{\tau }$ are the control derivatives.

#### 2.4.2. VTOL Model

For the VTOL (quad) mode (see [25]), no rotor faults are assumed in this paper where the challenge is to compensate wind disturbance and faults on the plane inputs simultaneously. Simultaneously handling rotor faults would compromise observability. However, wind disturbance is still present and the quadplane is trimmed and linearised for hover. The state-space model of the quad mode is given by the following:

(31) $$\dot{x}=A_{q}x+B_{q}u_{q}+B_{\mathrm{gq}}d_{g}$$

where $u_{q}={\left[F_{Zquad},M_{quad}\right]}^{T}$ and the state matrix of quad model is given by the following:

(32) $$A_{q}=\left[\begin{array}{ccccc} 0 & 0 & 0 & -g\cos(\theta^{*}) & 0\\ 0 & 0 & 0 & -g\sin(\theta^{*}) & 0\\ 0 & 0 & 0 & 0 & 0\\ 0 & 0 & 1 & 0 & 0\\ \sin(\theta^{*}) & -\cos(\theta^{*}) & 0 & U_{q}\cos(\theta^{*})+W_{q}\sin(\theta^{*}) & 0 \end{array}\right]$$

where $U_{q}$, $W_{q}$ are the trim conditions for the longitudinal and vertical speeds in the quad mode, which can be taken to be zero or small for numerical implementation. Likewise, the quad control matrix $B_{q}$ can be obtained by differentiation of *f* at $u=u^{*}\left(\mathrm{quad}\;\mathrm{mode}\right),$ which will be taken to be a hover condition for simplicity, assuming that climb rates have small variation with respect to the hover condition, which implies a very low drag at those climb velocities. The quad control matrix is given by the following:

(33) $$B_{q}=\left[\begin{array}{cc} 0 & 0\\ \frac{1}{m} & 0\\ 0 & \frac{l}{J_{y}}\\ 0 & 0\\ 0 & 0 \end{array}\right]$$

where m represents the mass and $J_{y}$ represents the moment of inertia about the pitch axis, and *l* is the arm length, which is assumed to be the same for all four rotors. The matrix $B_{\mathrm{gq}}$ is similarly obtained by differentiation of the aerodynamic disturbance vector $d_{1}={\left[F_{Xd},F_{Zd},M_{d}\right]}^{T}$ with respect to $d_{g}={\left[u_{g},w_{g},q_{g}\right]}^{T}$, which turns out to be equivalent to adding the wind gusts components to the velocity and angular rate channels [38].

#### 2.4.3. Transitioning Model

A gradual transitioning strategy is used and the transitioning state-space model under winds and potential plane command faults is given by the following:

(34) $$\dot{x}=A_{\mathrm{tr}}x+B_{\mathrm{tr}}u_{\mathrm{tr}}+B_{\mathrm{gt}}d_{g}+B_{p}f_{a}$$

The following transitioning control law before any disturbance rejection is adopted:

(35) $$u_{\mathrm{tr}}=\left(\frac{u-u_{\min}}{u_{\max}-u_{\min}}\right)u_{p}+\left(1-\frac{u-u_{\min}}{u_{\max}-u_{\min}}\right)u_{q}$$

where $u_{\min}=$2 m/s and $u_{\max}=$10 m/s are taken here as the minimum and maximum longitudinal cruise speeds of the transition mode after plane to quad (P2Q) or quad to plane (Q2P) transitions. During transitioning, plane commands $u_{p}={\left[\eta ,\tau \right]}^{T}$ and quad commands $u_{q}={\left[F_{Zquad},M_{quad}\right]}^{T}$ are both used. Using this gradual transitioning strategy, the quadplane is only controlled by the quad control inputs vector $u_{q}$ when axial speed satisfies $u<u_{\min}$ and only controlled by the plane control inputs vector $u_{p}$ when $u>u_{\max}$. Note that this transitioning strategy also allows a decreased lift during the Q2P transition and increased lift during the P2Q transition, using a linear approximation to the lift force as a function of velocity.

**Figure 3:**
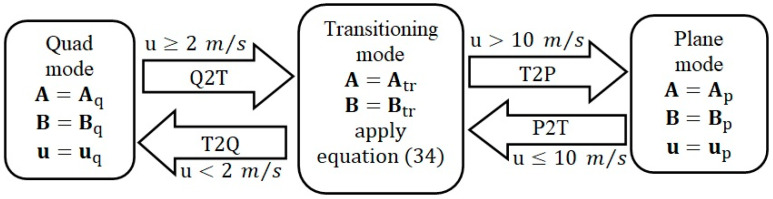
Quadplane gradual transitioning hybrid control system.

The Q2P transition is triggered when the speed threshold $u_{min}$ is exceeded. This threshold is reached at a time defined by the desired axial speed profile. The quad commands are then progressively turned off while the plane commands are progressively turned on using the control law of Equation (35). Likewise, the plane to quad transition is triggered when the cruise speed falls below $u_{max}$. The plane commands are then progressively turned off while the quad commands are progressively turned on. The gradual transitioning logic is described in Figure 3.

The variable matrices $A_{\mathrm{tr}}$, $B_{\mathrm{tr}}$ and $B_{\mathrm{gt}}$ are trimmed by progressively increasing the trimmed cruise speed from 2 m/s to 10 m/s for the transition from quad to plane mode and progressively decreasing them from 10 m/s to 2 m/s for the transition from plane to quad mode. A numerical method based on sequential quadratic programming using Matlab’s Trim command is used to update the trim conditions between the operating speeds of the plane and quad modes, even though the calculations can be verified by hand as the trim condition. A lookup table is used to store the $A_{\mathrm{tr}}$ and $B_{\mathrm{tr}}$ matrices for 20 evenly spaced speed values between 2 m/s and 10 m/s, and a simple 1D interpolation is used when the speeds are between any two consecutive gradual trim speed values from the lookup table. The compensation of any wind disturbances is performed in the same way as for the other modes. The compensation of any plane input faults can be performed in the same way as for the plane mode.

### 2.5. Atmospheric Turbulence Model

Wind models for observer-based control papers were often found to be simple trigonometric functions, which do not provide sufficient realism. A Dryden wind turbulence model is used here to provide the wind velocities $u_{g},$ $w_{g}$ and the wind angular rate effect $q_{g}$ that the UAV encounters under realistic flight conditions. The wind model was developed and validated by comparison against the Dryden model output block of the MATLAB UAV blockset. The disturbance terms $u_{g},$ $w_{g}$ and $q_{g}$ are related to the altitude. For altitudes *h* of up to 1000 ft, turbulence scales and intensities are given by:

(36) $$\{\begin{array}{l} 2L_{w}=h\\ L_{u}=\frac{h}{{\left(0.77+0.000823\right)}^{1.2}}\\ \sigma_{w}=0.1W_{s}\\ \sigma_{u}=\frac{\sigma_{w}}{{\left(0.77+0.000823\right)}^{0.4}} \end{array}$$

where $L_{w}$ and $L_{u}$ represent the vertical and longitudinal turbulence scale lengths in ft, respectively, $\sigma_{w}$ denotes the vertical turbulence intensity in ft/s and $W_{s}$ is the average wind speed intensity, representing the level of severity assumed in the turbulence level.

The longitudinal and lateral intensities $\sigma_{u}$ and $\sigma_{w}$ are equal in this model:

(37) $$\sigma_{u}=\sigma_{w}$$

The wind turbulence velocities and angular rates terms are then obtained by applying a white Gaussian noise input to the transfer functions of Table 1, which are in agreement with the MIL-HDBK-1797B standard.

**Table 1:** Transfer functions for velocity spectra [36].

Continuous Dryden Filter	MIL-HDBK-1797B
$H_{u}\left(s\right)$ Transfer function for the longitudinal wind velocity	$\sigma_{u}\sqrt{\frac{2L_{u}}{\pi V}}.\frac{1}{1+\frac{L_{u}}{V}s}\;\;\;\;\left(38\right)$
$H_{w}\left(s\right)$ Transfer function for the vertical wind velocity	$\sigma_{w}\sqrt{\frac{2L_{w}}{\pi V}}.\frac{1+\frac{2\sqrt{3}L_{w}}{V}s}{{\left(1+\frac{2L_{w}}{V}s\right)}^{2}}$ (39)
$H_{q}\left(s\right)$ Transfer function for the pitch rate due to wind gusts	$\begin{array}{c} \frac{\pm \frac{s}{V}}{\left(1+\left(\frac{4b}{\pi V}\right)s\right)}H_{w}\left(s\right)\;\;\;\left(40\right) \end{array}$

## 3. State and Perturbation Observers

### 3.1. State Observer

A conventional Luenberger observer is used to estimate the longitudinal state variables. Process and measurement noise are not considered in this paper, as sensor noise and model uncertainty are not the issue under consideration. A Kalman filter could be designed to handle those issues if necessary. The overarching aim of the paper is to estimate the wind gusts and actuator faults. The Luenberger state observer can estimate the full state vector in the disturbance-free case, as long as the number of measurements is sufficient to guarantee observability. This is the case when measuring speed and altitude alone. Although the full state vector can be measured here for simplicity, because the UAVs under consideration are typically equipped with the necessary inertial measurement units, GPS, pressure sensing and sensing errors are not the focus.

The state observer equations are given:

(41) $$\dot{\hat{x}}=A\hat{x}+Bu+L\left(y-\hat{y}\right)$$

$$\hat{y}=C\hat{x}$$

It is well known that the estimation error $e=x-\hat{x}$ of this observer converges to zero with exponential convergence rate **L**. The matrices **A** and **B** in Section 3, Section 4, Section 5 and Section 6 are equal to $A_{p},B_{p}$ in the plane mode, $A_{q},B_{q}$ in the quad mode and $A_{\mathrm{tr}},B_{\mathrm{tr}}$ in the transition modes, respectively. Wind disturbances and faults affect the measured output y.

### 3.2. Unknown Input Observer for Wind Perturbation Estimation

An unknown input observer is employed to estimate the external wind gusts perturbation vector, which is defined as $d_{g}={\left[u_{g},w_{g},q_{g}\right]}^{T}$ assuming perfect sensor measurements without sensor noise, which is not the focus of the paper. The components of $d_{g}$ represent the wind gust effects on the axial and vertical velocities and on the pitch rate.

The state-space model of a linearised system with wind as the only disturbance can be written as

(42) $$\dot{x}=Ax+Bu+B_{g}d_{g}$$

By defining the term $d_{1}=B_{g}d_{g}$, the ability to estimate $d_{1}$ is proven before the estimation of $d_{g}$. We introduce the following unknown input observer equation:

(43) $${\hat{d}}_{1}=z+k\;\hat{x}$$

(44) $$\dot{z}=-k\left({\hat{d}}_{1}+A\hat{x}+Bu\right)$$

where ${\hat{d}}_{1}$ is the estimate of $d_{1}$, z is an auxiliary input that is defined to emulate body accelerations and circumvent the need for acceleration measurements and k is the scalar gain of the perturbation observer. The estimation error is defined as:

(45) $${\tilde{d}}_{1}=d_{1}-{\hat{d}}_{1}$$

Assuming a bias type fault model, which will only be valid for slowly varying or piecewise constant disturbances, we have the following:

(46) $${\dot{\tilde{d}}}_{1}=-{\dot{\hat{d}}}_{1}$$

A convergence proof is simpler to establish using the well-known property of the Luenberger observer, which makes $\hat{x}$ converge to x at the steady state. In practice, the state observer gain must also be sufficiently high to ensure that the state observer converges faster than the disturbance observer, which is feasible. After the state observer has converged, the disturbance observer equations can then be written in the following form:

(47) $$\begin{array}{ll} {\dot{\tilde{d}}}_{1} & =-\dot{\;z}-k\;\dot{\hat{x}}\approx -\dot{\;z}-k\;\dot{x}\\ & =k\left({\hat{d}}_{1}+A\hat{x}+Bu\right)\\ & \;-\left(A\hat{x}+Bu+d_{1}\right)\\ & =-k\left(d_{1}-{\hat{d}}_{1}\right)=-k{\tilde{d}}_{1} \end{array}$$

Therefore, ${\hat{d}}_{1}$ converges exponentially to $d_{1}$ with k > 0 under the above conditions.

The wind gust disturbance vector ${\hat{d}}_{g}$ can then be obtained using the Moore pseudoinverse as follows:

(48) $${\hat{d}}_{g}=B_{g}{}^{†}{\hat{d}}_{1}={\left(B_{g}{}^{T}B_{g}\right)}^{-1}B_{g}{}^{T}{\hat{d}}_{1}$$

where the dagger symbol † denotes the Moore-Penrose pseudoinverse.

### 3.3. Auxiliary Variable Observer with Exponential Convergence Rate (AVOECR) for Wind Disturbance and Fault Estimation

The wind disturbance and fault observation model can be written more generally as

(49) $$d_{1}=B_{g}d_{g}+B\;f_{a}=B_{\mathrm{ext}}d_{\mathrm{ext}}$$

where $f_{a}$ is an actuator fault vector, and

(50) $$B_{\mathrm{ext}}={\left[B_{g},B]\;,\;d_{\mathrm{ext}}=[d_{g}^{T},f_{a}^{T}\right]}^{T}$$

However, it is unfortunately not possible to obtain the full rank of $d_{g},f_{a}$ by direct pseudoinversion of $d_{1}$, because the rank condition for pseudoinversion is not satisfied due to the zeros in the matrices $B_{g},B$.

It is therefore convenient to formulate a problem with only one fault component and two of the three wind components to make the problem solvable. Numerical simulations show that the most important elements of **d** are $u_{g},w_{g}$, both of which have low and high-frequency components. The term $q_{g}$ is simply a high-frequency noise term similar to pitch-rate measurement noise, for which exact compensation is less crucial as it is naturally compensated using the robustness of more stabilising optimal controllers such as LQR. The control design choice is therefore to only use the observer-based correction to compensate for the effects of $u_{g},w_{g}$ and one actuator fault, taken here to be an elevator fault without loss of generality. The effect of $q_{g}$ is still included in the simulation, but is not estimated or compensated; the observable parts of $B_{\mathrm{ext}}$ and $d_{\mathrm{ext}}$ are defined as

(51) $$B_{o-\mathrm{ext}}={\left[B_{g}^{1:2},B^{1}],d_{o-\mathrm{ext}}=[u_{g},w_{g},f_{\eta }\right]}^{T}$$

where $B_{g}^{1:2}$ denotes the first two columns of $B_{g}$, $B^{1}$ denotes the first column of **B** and ${\hat{d}}_{1}=B_{o-\mathrm{ext}}\;{\hat{d}}_{o-\mathrm{ext}}$, and $f_{\eta }$ represents the elevator fault.

It is then possible to estimate both wind gust velocities and the fault using

(52) $${\hat{d}}_{o-\mathrm{ext}}=B_{o-\mathrm{ext}}{}^{†}\;{\hat{d}}_{1}={\left(B_{o-\mathrm{ext}}{}^{T}B_{o-\mathrm{ext}}\right)}^{-1}B_{o-\mathrm{ext}}{}^{T}\;{\hat{d}}_{1}$$

Equation (52) allows for a real-time simultaneous estimation of an actuator fault and of the two wind velocity components $u_{g},w_{g}$ along the x and z body axes.

The extension of the unknown input observer of Equations (43) and (44) from Section 3.2 to the combined wind velocities and fault estimation using Equation (52) will be named auxiliary variable observer with exponential convergence rate (AVOECR).

## 4. Observer-Based LQR with Disturbance and Fault Rejection

### 4.1. Reference Following LQR

For simplicity, we first present LQR for the fault-free and undisturbed case. A non-zero setpoint tracking linear quadratic regulator (LQR) is used to determine the optimal state feedback controller u(x) that minimises the following quadratic cost function:

(53) $$J=\int_{0}^{\infty }(e^{T}Qe+u^{T}Ru)\mathrm{dt}$$

subject to $\dot{x}=Ax+Bu$,

where $e=x-x_{c}$ is the trajectory tracking control error and $x_{c}$ is the desired state. The **A** and **B** matrices are mode dependent and are taken in this section to be equal to $A_{p},B_{p}$ in the plane mode, $A_{q},B_{q}$ in the quad mode, $A_{\mathrm{tr}},B_{\mathrm{tr}}$ if gradual mode transitions are used and the control input is $u=u_{p}$ in the plane mode, $u=u_{q}$ in the quad mode and $u=u_{\mathrm{tr}}$ from Equation (35) is used in the transition mode.

The desired output will be denoted $y_{c}$. For non-zero reference following, the dimension of the output vector $y=C_{y}x$ is assumed to be equal to the dimension of the control input vector, which is the case, for example, when speed and altitude are controlled using the throttle and elevator with an adequate choice of $C_{y}$. Any desired states other than the outputs would still be stabilised and controlled to zero during the manoeuvre.

The optimal control input is given:

(54) $$u_{\mathrm{LQR}}=-Ke+u_{c}$$

where the optimal gain is given by

(55) $$K=R^{-1}B^{T}p$$

and p solves the steady state algebraic Riccati equation:

(56) $$pA-pBR^{-1}B^{T}p+A^{T}p+Q=0$$

The term $u_{c}$ is necessary to allow for the simultaneous tracking of non-zero references and is given by

(57) $$u_{c}=M_{u}y_{c}$$

and the desired state satisfies

(58) $$x_{c}=M_{x}y_{c}$$

with

(59) $$\left[\begin{array}{c} M_{x}\\ M_{u} \end{array}\right]={\left[\begin{array}{cc} A & B\\ C_{y} & 0_{2\times 2} \end{array}\right]}^{-1}\left[\begin{array}{c} 0_{5\times 2}\\ I_{2\times 2} \end{array}\right]$$

This state feedback controller is not part of the novelty, but the combination with the proposed disturbance and fault observer is a contribution of this paper.

### 4.2. LQR with Observer-Based Active Disturbance and Fault Rejection

**Figure 4:**
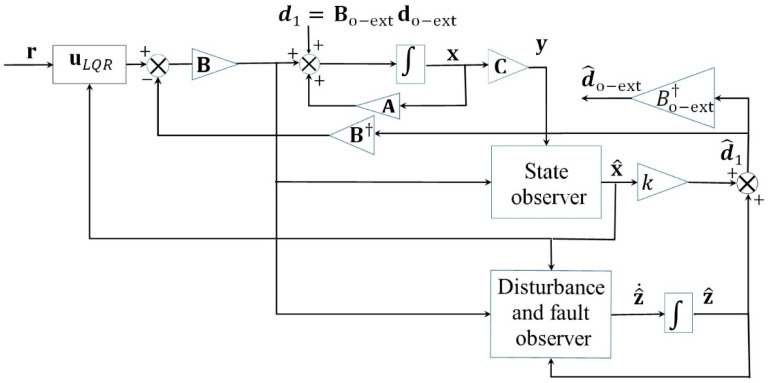
Block diagram of the disturbance and fault rejection AVOECR-LQR control loop.

Without loss of generality, LQR is taken as a benchmark controller to be enhanced by the observer. The unknown input observer is used to estimate and compensate the wind disturbance alone or its velocity components together with an actuator fault. Figure 4 shows the observer-based control system block diagram for the combined wind and fault rejection. The recovery control law for both cases of wind rejection (all three components) or combined active wind and fault rejection (two wind components + fault) is given:

(60) $$Bu=Bu_{\mathrm{LQR}}-{\hat{d}}_{1}$$

(61) $$u=u_{\mathrm{LQR}}-B^{†}{\hat{d}}_{1}$$

This control law either eliminates all three components of the wind disturbance ($u_{g},w_{g},q_{g}$) over time if Equation (48) is used, or it suppresses the effects of the two velocity components of the wind and elevator fault ($u_{g},w_{g},f_{\delta }$) if Equation (52) is used. The estimation error still converges to zero because the observer convergence proof is independent from the controller. The rate of convergence of the observer increases with observer gain *k* and the one for LQR tracking can be tuned using the **Q** and **R** matrices of LQR. Crucially, high observer gains do not imply high controller gains using this approach.

## 5. Numerical Simulation Analysis

As previously explained, an aerosonde type quadplane UAV is assumed in this numerical simulation section, where Matlab 2021a was used together with Simulink to develop the UAV model. The UAV mass is 13.5 kg, the moment of inertia about the pitch axis is 1.135 kg.m^2^, the wingspan is 2.9 m and the arm length of the rotors is 0.46 m. The operating condition is a speed of 30 m/s and a more complete set of aerodynamic parameters is given in [29,38]. The thrust coefficient of the quad motors is not directly used since the control inputs are thrust and moment, and the maximum thrust of each rotor is 50 N. Note that even though both elevator and throttle faults scenarios were successfully tested during both the plane and transition modes, this numerical simulation section is focused on the case of an elevator fault under windy conditions during the plane mode for paper length considerations. The same approach can be used to handle additive throttle faults or any single plane actuator faults during transitioning too. As previously explained, throttle and elevator faults cannot be simultaneously handled with wind rejection, as this would compromise observability.

### 5.1. Observer-Based Wind Disturbance Rejection

In this subsection, the proposed AVOECR observer is combined with LQR, as shown in Figure 4, for wind disturbance rejection in the fault-free case. Comparisons against other observers and controllers are not included in this section because the main benefit of our approach will be more evident in the case of combined wind and fault recovery. For simplicity, we first present the results and validate the proposed approach in the case of fault-free wind disturbance rejection.

#### 5.1.1. Wind Observer Estimation Accuracy

**Figure 5:**
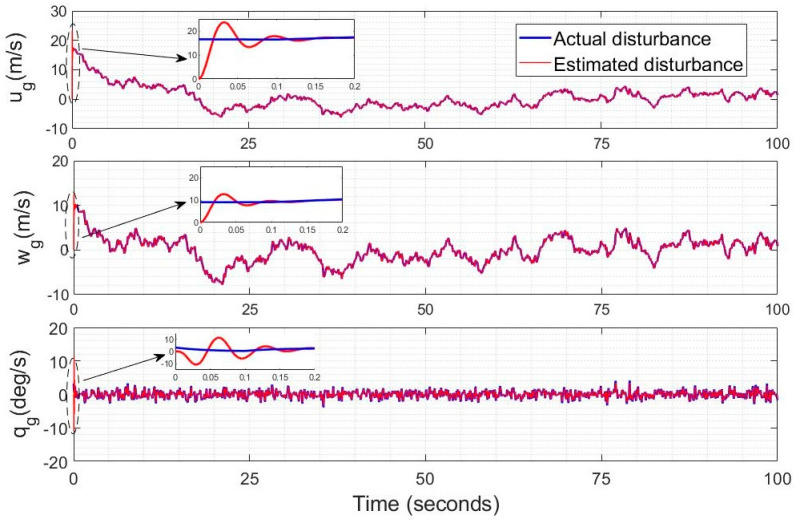
Wind perturbation estimation using the unknown input observer.

The wind disturbance follows a Dryden model with an average wind intensity $W_{s}$ of 5 m/s; the perturbation observer is shown in the zoomed intervals of Figure 5 to track all components of the wind perturbation vector rapidly with a perturbation observer gain *k* = 10. The terms $u_{g},$ $w_{g}$ are tracked slightly more rapidly than $q_{g}$. The wind gusts velocities have their usual characteristic low- and high-frequency components.

#### 5.1.2. Effect of the Observer Gain on Wind Disturbance Rejection

**Figure 6:**
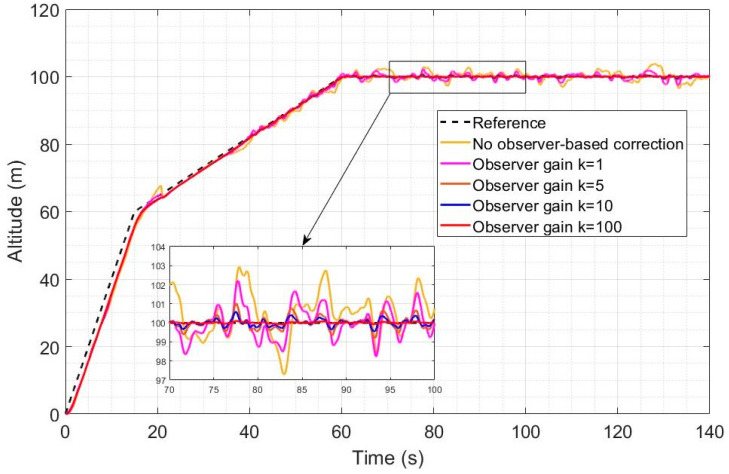
Altitude reference tracking under a high wind intensity for different values of the perturbation observer gain.

**Figure 7:**
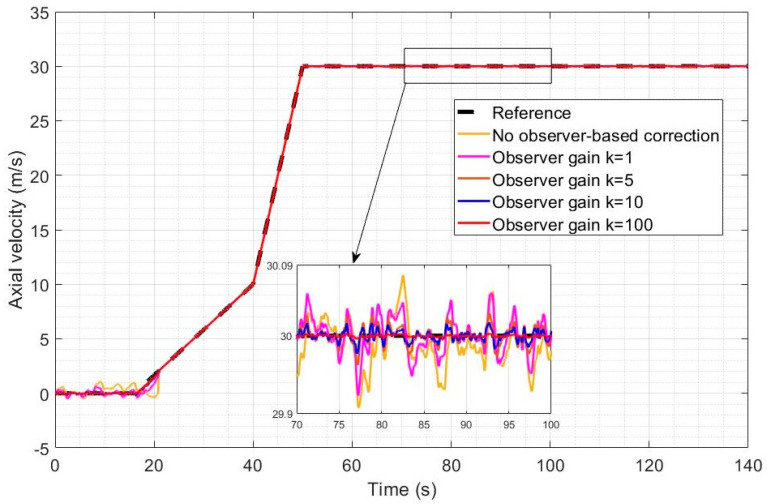
Axial speed reference tracking under a high wind intensity for different values of the perturbation observer gain.

Figure 6 and Figure 7 represent the altitude and speed reference tracking performance in the case of a relatively high wind intensity of $W_{s}$ = 5 m/s. In Figure 6, altitude reference tracking performance is shown to be significantly enhanced by a factor of 2 to 4 using LQR with observer-based compensation (Equation (61), compared to LQR without observer-based compensation (Equation (54)). Higher observer gains are also shown to lead to improved disturbance rejection when the observer-based recovery controller of Equation (61) is used. A 3 m overshoot is also observed without observer compensation after the transition and the higher observer gains fully remove the overshoot. In Figure 7, a similar level of improvement can be observed in the tracking of the desired speed using the observer-based disturbance rejection controller with a more pronounced enhancement in the reference speed tracking when higher observer gains are used. The desired speed profile was designed to ensure that a velocity of 2 m/s is obtained in the quad mode before switching to the Q2P transition mode. Transitioning directly from 0 m/s would have caused the issue of frequent switching because the controller, despite its high efficiency, cannot keep axial velocity exactly zero in the quad mode.

**Figure 8:**
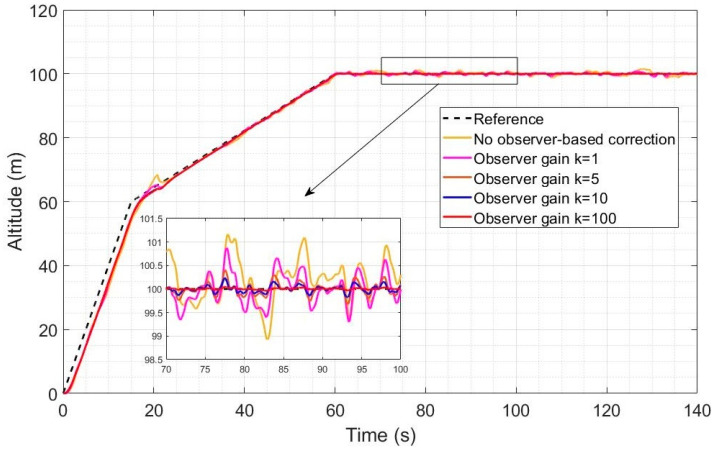
Altitude reference tracking under a low wind intensity for different values of the perturbation observer gain.

**Figure 9:**
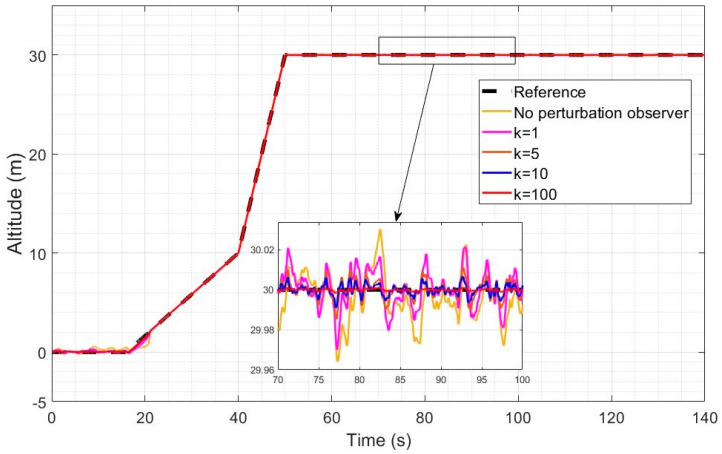
Axial peed reference tracking under a low wind intensity for different values of the perturbation observer gain.

Figure 8 and Figure 9 represent the altitude and speed reference tracking performance with a relatively low wind intensity of $W_{s}$ = 2 m/s. In Figure 8, both controllers are shown to provide accurate altitude reference tracking, but the observer-based controller still further improves the already good performance of LQR. Increasing the observer gain also leads to improved disturbance rejection as expected. In Figure 9, both controllers achieve very good reference speed tracking accuracy under this low wind intensity, but the observer-based compensation still leads to improved disturbance rejection and tracking performance.

**Figure 10:**
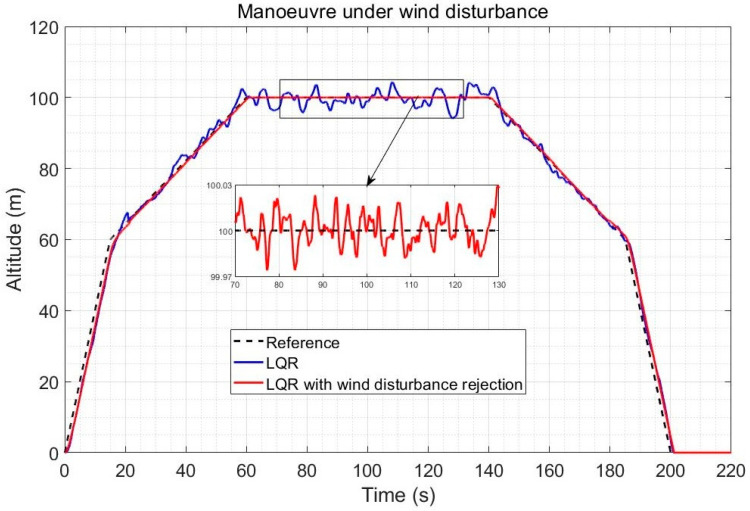
Altitude response comparison during a climb-cruise-land trajectory under an average wind intensity of 5 m/s.

The integrated absolute error (IAE) metrics obtained for the simulations of Figure 7, Figure 8, Figure 9 and Figure 10 are shown in Table 2. The altitude and velocity IAE are both substantially and increasingly reduced by increasing the gains, and the difference is more pronounced with the higher intensity of 5 m/s. With the highest observer gain, the IAE is indeed reduced by a factor of 9.82 for the altitude and a factor of 2.41 for the velocity, compared to control without an observer.

#### 5.1.3. Observer-Based Trajectory Tracking Control under Wind Gusts

**Figure 11:**
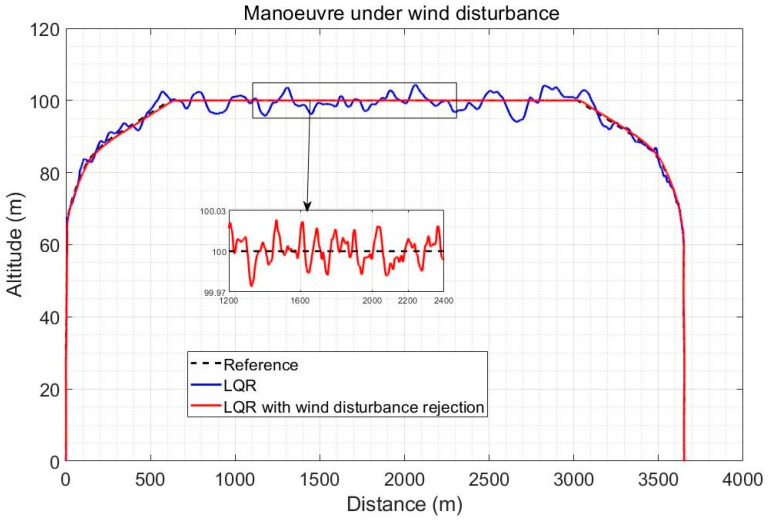
Trajectory tracking comparison for a climb-cruise-land quadplane trajectory under an average wind intensity of 5 m/s.

The altitude response for a full quadplane vertical climb, cruise and vertical landing trajectory is shown in Figure 10 in the case of a severe 5 m/s wind intensity and using the gradual transitioning strategy between the quad and plane modes between 2 m/s and 10 m/s forward speeds. The speed reference change was applied shortly before the transitioning. The improvement obtained by observer-based compensation of the wind disturbance (with *k*= 100) is shown to be significant, particularly during the plane and transitioning stages. Note that the x axis of Figure 10 is time, not distance, and the linear increase in altitude is initially in the vertical direction as shown in Figure 11.

Figure 11 illustrates the fact that both controllers perform the vertical climb accurately, but that the observer-based compensation provides a significantly finer altitude reference tracking during the plane mode, with tracking errors in centimetres compared to up to 5 m error using LQR alone. In practice, sensor noise (which is not the focus of this paper) will slightly degrade the accuracy, but it is expected that the observer-based controller will still not exceed 1 m error, while standard LQR already exceeds 5 m error without the noise effects, which would affect both controllers similarly. The improvement using observer-based disturbance rejection is also significant during the transitioning stages.

### 5.2. Observer-Based Control with Active Combined Wind and Fault Rejection

#### 5.2.1. Observer-Based Combined Wind Disturbance and Fault Estimation

In this section, our AVOECR observer is compared to three observers, which are inspired by the literature, but adapted to the system model and are described as follows:

The first alternative observer, which will be called output error integral observer (OEIO) is inspired from [30,31]; in these references, however, the observer was only applied to the altitude and pitch rate channels, respectively. This is extended here to perturbations affecting the full state using

(62) $$\begin{array}{c} \dot{\hat{x}}=A\hat{x}+Bu+{\hat{d}}_{1}\\ {\dot{\hat{d}}}_{1}=-k\left(\hat{y}-y\right) \end{array}$$

where *k* is an observer gain, with y=Cx and $\hat{y}=C\hat{x}$ and **C** = $I_{5\times 5}$ for simplicity in the observer, with no sensor noise due to the focus on perturbations.

The second observer used for comparison is an auxiliary variable-based sliding mode observer (AVSMO), which can be seen as a special case of the observer used in [40] with a nonlinear but smooth tanh sliding surface and adapted to our model. It is given by

(63) $$\begin{array}{c} \dot{z}=-k\;\dot{\hat{x}}-k\;\tanh\left(a\left(z+K\;\hat{x}\right)\right)\\ {\hat{d}}_{1}=z+k\;\hat{x} \end{array}$$

where *k* is an observer gain, a a smoothing parameter to alleviate chattering and oscillations about $z+k\;\hat{x}$, which is taken here to be equal to 0.5 and **tanh** applies a four-quadrant tangent hyperbolic function (instead of sign functions) to all elements of the vector $a\left(z+k\;\hat{x}\right)$. The auxiliary variable vector **z** is similar to the one used in our approach but is designed to give the convergence properties of a sliding mode observer.

The third and last observer used for comparison will be termed rate and acceleration measurements-based observer (RAMO) and is inspired from [39], but simplified and adapted to our application. It is given by

(64) $${\dot{\hat{d}}}_{1}=\;k\left[\dot{x}-{\dot{\hat{x}}}_{d_{1}=0}-{\hat{d}}_{1}\right]$$

where *k* is an observer gain. However, a limitation of this approach is that $\dot{x}$ is typically assumed to be measured using rate and acceleration sensors as indirect perturbation measurements, which is costly, and the acceleration measurements are noisy in practice unless complexity is increased using complementary filtering or an equivalent approach.

**Figure 12:**
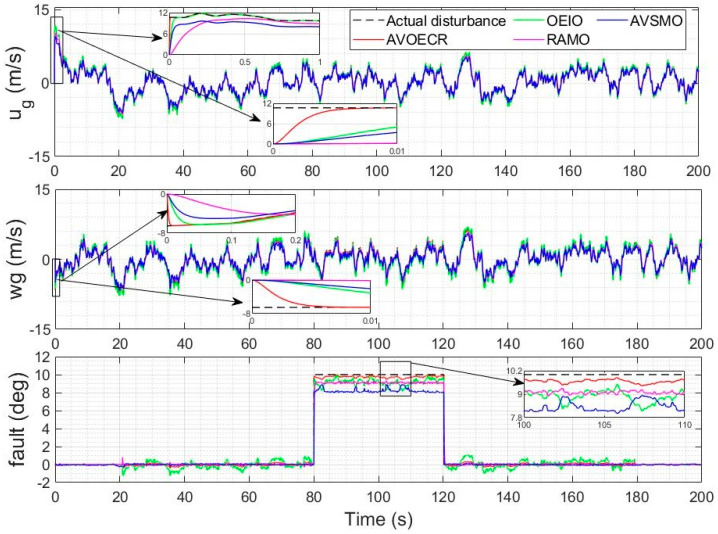
Actual and estimated wind velocities and actuator fault using observer-based compensation with all observers (equivalent gains comparison).

**Figure 13:**
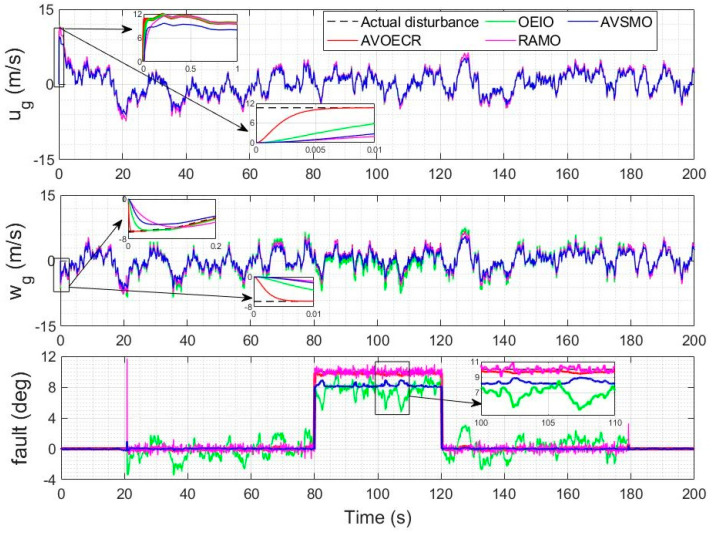
Actual and estimated wind velocities and actuator fault using observer-based compensation with all observers (allowing higher gains for alternative controllers).

The actuator fault scenario under consideration in this section is an intermittent 10 degrees elevator fault between *t* = 80 s and *t* = 120 s, using a Dryden model for the wind velocities with a 5 m/s average wind intensity and the observer-based controller of Section 4.2. Scalar observer gains are used in all observers to make a fair comparison simpler. In Figure 12, all observers are compared to our proposed AVOECR approach, using the same observer gain of *k* = 100, except for AVSMO, for which gains are reduced to alleviate the chattering. For this scenario, the AVOECR clearly outperforms all other observers with finer accuracy for the estimation of both wind velocities and the fault. The OEIO observer is second best for the wind velocities estimation and performs slightly better than the RAMO when the fault is active, but increases fault estimation error when the fault is not present. All observers perform well in terms of wind perturbation estimation, but there is a significant error on the fault estimation compared to the AVOECR. Figure 13 shows a similar comparison where all alternative observers are allowed higher gains but keep *k* = 100 with AVOECR. Increasing gains of the three alternative observers causes a degradation in fault estimation. With the RAMO observer, increasing gains reduces the bias error on fault estimation, but the estimation remains noisy compared to the AVOECR, with large spikes at the mode transition times. With OEIO, higher gains cause significantly higher oscillations in the fault estimation. With AVSMO, increasing gains slightly reduces oscillations but does not significantly change the bias. Smaller spikes are observed at the transition times. Increasing the AVSMO gains even further cause the estimation performance to be too significantly degraded with chattering and spikes, so it was not included in the figure. In Table 3, the AVOECR clearly outperforms all three alternative observers in terms of IAE for the combined fault and wind velocities rejection scenario, whether equivalent gains are used (with improvement by a factor of 2.28 to 44.7, depending on the perturbation component and observer) or when alternative observers are allowed higher gains. Increasing the gains of alternative observers reduces the IAE of the wind perturbations but increases the fault IAE, especially with the OEIO observer.

#### 5.2.2. Observer-Based Controllers Comparison under Simultaneous Actuator Fault and Wind Disturbance

##### 5.2.2.1. LQR with Observer-Based Disturbance and Fault Rejection

LQR control is used as a benchmark controller in this section with $Q=I_{5\times 5}$ and R= diag(0.0011, 0.001), and uses the scenario of Figure 12, where all observers have the same observer gain of 100 (except AVSMO which uses a gain of 50 to avoid chattering), to make the comparison favourable for alternative observers that did not exhibit unusual spikes or substantial noise in this case.

**Figure 14:**
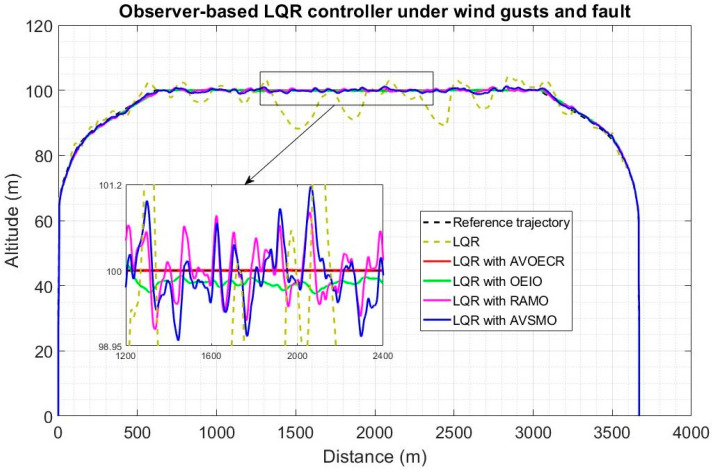
Quadplane trajectory tracking comparison under a 5 m/s wind intensity with an elevator fault between 80 s and 120 s, with a LQR benchmark controller.

In Figure 14, four observer-based controllers using the architecture of Figure 4 and Equation (61) are compared for a typical quadplane flight profile using the same four different observers (AVOECR, OEIO, RAMO and AVSMO) elevator fault and wind perturbation compensation. The fault and wind gusts scenario is the same as in Section 5.2.1. With all four observers, observer-based control significantly outperforms the LQR controller without observer compensation. However, the LQR-AVOECR based fault and disturbance compensation clearly outperforms all other approaches in terms of path following accuracy, particularly when the fault is present between 80 s and 120 s (during the plane mode). The OEIO performs well but less accurately than AVOECR, the AVSMO and RAMO observers have error peaks at 1 m and 0.8 m, respectively, and the maximum altitude error deviation using LQR without any observer-based compensation exceeds 10 m. The transitioning stages occur between 20 s and 40 s and again between 140 s and 160 s. The wind with 5 m/s average intensity is present during the whole simulation and is much more efficiently rejected using the observer-based compensation approaches, with notably high performance using AVOECR.

The altitude and velocity IAE results for the observer-based LQR controllers with disturbance and fault rejection are summarised in Table 4, with a comparison against standard setpoint tracking LQR. With AVOECR, the altitude IAE and velocity IAE are reduced by a factor of 4.41 and 4.55, respectively, compared to LQR without observer correction. Compared to the best alternative observer (OEIO), AVOECR reduces the altitude IAE and velocity IAE by 30% and 32%, respectively.

##### 5.2.2.2. Observer-Based H∞ Control

To demonstrate the generality of the principle of performance enhancement compared to a benchmark controller, a similar comparison is performed by taking H∞ control [41] as a benchmark controller, with $u=u_{H\infty }-B^{†}\;{\hat{d}}_{1}$ and using all four observers to estimate ${\hat{d}}_{1}$. The H∞ controller is designed to achieve a tradeoff between a sensitivity function **S**, which needs to be attenuated at low frequencies to reject slow disturbance effects, and a complementary sensitivity function **T**, which needs to be attenuated as high frequencies to mitigate noise (high wind frequencies) effects. The two conditions cannot be satisfied at the same frequency because **S + T =** I. Matlab command mixsyn is used for the mixed sensitivity design to determine the feedback **u**(s) that satisfies

(65) $$\underset{u\left(s\right)}{\min}\|{\left[\begin{array}{c} W_{S}S\left(s\right)\\ W_{T}T\left(s\right) \end{array}\right]\|}_{\infty }$$

where $\|\;\|{}_{\infty }$ is the infinity norm and the weighting functions of **S** and **T** are tuned to shape the loops and mitigate output oscillations due to disturbances (peak singular value = 0.73) linked to the low and high wind frequencies. They are given by

(66) $$\begin{array}{c} W_{S}\left(s\right)=\frac{0.5s+4}{s+0.08}\\ W_{T}\left(s\right)=\frac{10s+120}{s+240} \end{array}$$

This is done for the same flight profile used for LQR and assuming the same fault and wind perturbations scenario. The optimisation of the H∞ controller tuning is complex and is not our focus here. The objective is to demonstrate improvement by observer-based compensation, given a H∞ controller setting.

**Figure 15:**
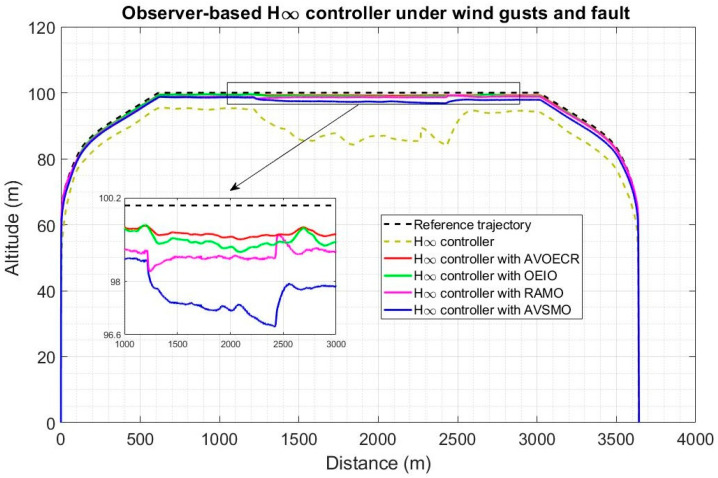
Quadplane trajectory tracking comparison under a 5 m/s wind intensity with an elevator fault between 80 s and 120 s, with a $H_{\infty }$ benchmark controller.

In Figure 15, H∞ control exhibits higher error biases but less oscillations compared to LQR (tuning for robustness). The observer-based compensation significantly enhances trajectory tracking accuracy with all four observers. The AVOECR-H∞ approach clearly provides the best observer-based compensation and trajectory tracking, particularly when the fault is present. This is done with a higher bias error, but all approaches appear to reduce oscillations when the fault is active between 80 s and 120 s. The maximum altitude error for this scenario is 1 m using the AVOECR observer, 1.45 m and 1.65 m with the OEIO and RAMO observers, respectively, and 3.1 m with the AVSMO observer. Without observer-based compensation, the maximum deviation is about 10 m on average, with a peak of 15 m when the fault is present. Therefore, observer-based compensation not only enhances performance but also safety in this scenario.

The altitude and velocity IAE results for the observer-based H∞ controllers with disturbance and fault rejection are summarised in Table 5, with a comparison against H∞ trajectory tracking. Using AVOECR, the altitude IAE and velocity IAE are reduced by 8% and 28%, respectively, compared to the second-best observer (OEIO-H∞). Compared to standard H∞ control without an observer, the altitude IAE and velocity IAE are reduced by factors of 3.41 and 3.96, respectively.

The AVOECR was shown to outperform all other observers for trajectory tracking with wind disturbance and fault rejection, using either LQR or H∞ feedback as benchmark controllers. However, it is important to verify that the observer-based compensation does not increase control inputs to inadmissible levels.

### 5.3. Control Effort Evaluation

For simplicity and paper length considerations, the control effort evaluation is focused on the comparison between conventional LQR trajectory tracking and our proposed AVOECR observer-based disturbance rejection control, with LQR as a benchmark controller. The best tracking performance for the climb-cruise-land manoeuvre scenario under consideration was obtained using this observer-controller combination. The aim is to demonstrate that the added control effort needed to implement the observer-based compensation is small and clearly admissible, within actuator limitations.

#### 5.3.1. Control Effort Comparison under Wind Gusts

**Figure 16:**
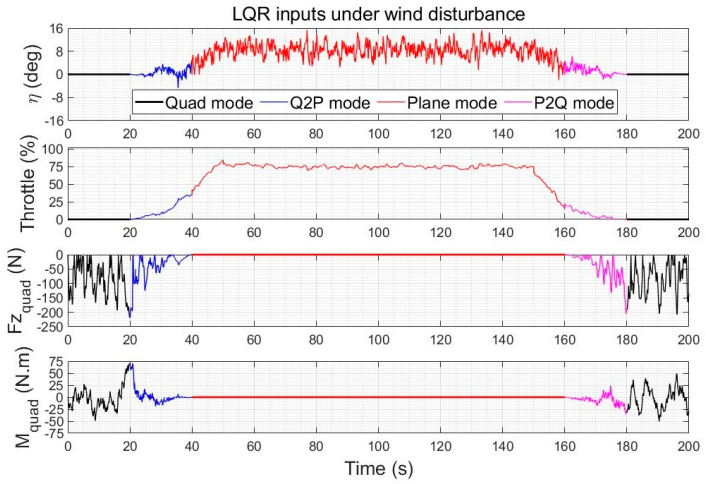
Quadplane control inputs using LQR during a climb−cruise−land manoeuvre under winds (no actuator fault).

**Figure 17:**
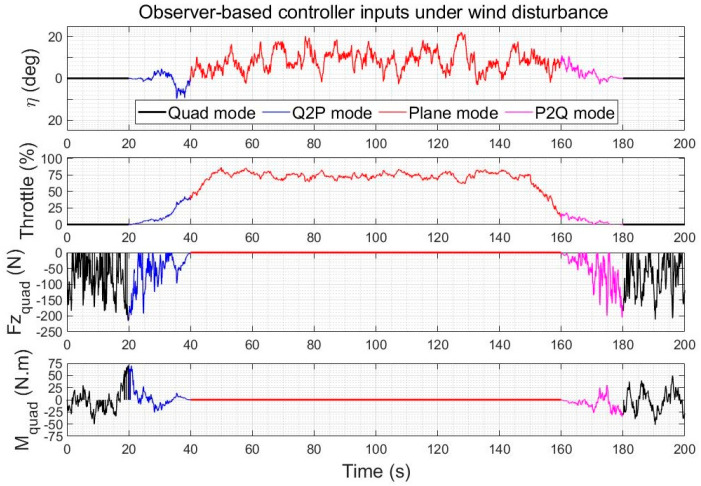
Quadplane control inputs using the observer-based wind disturbance rejection control during a climb-cruise-land manoeuvre under winds (no actuator fault).

The control inputs of the quadplane using reference following LQR and using the augmented LQR with observer-based wind disturbance rejection are shown in Figure 16 and Figure 17, respectively, for the above two climb-cruise-land flight of Figure 10 and Figure 11. The wind disturbance observer adds a contribution to the control inputs, but the increase in energy consumption is low compared to the pronounced improvement in trajectory tracking accuracy, rapidity and robustness to winds. The high-frequency components of the control inputs were verified to be feasible with both controllers. They are mainly used to compensate the higher frequency components of the wind gusts. The disturbance rejection controller compensates more efficiently for the lower frequencies of the wind gusts.

#### 5.3.2. Control Effort Comparison under Wind Gusts and Faults

**Figure 18:**
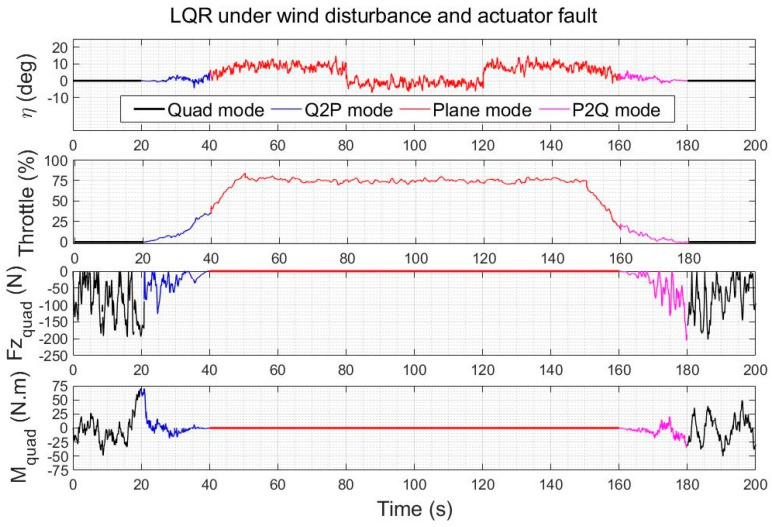
Quadplane control inputs using LQR during a climb−cruise−land manoeuvre under winds and an elevator fault between 80 s and 120 s.

**Figure 19:**
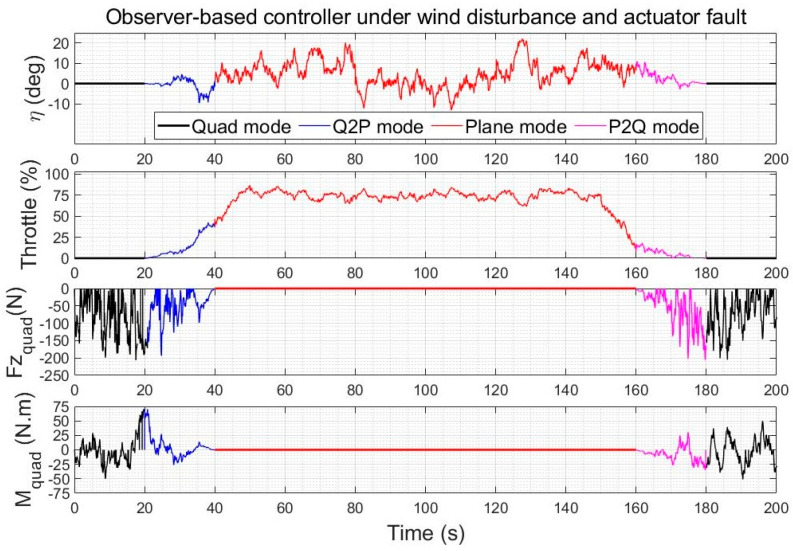
Quadplane control inputs using the observer−based controller during a climb−cruise−land manoeuvre under winds and an elevator fault between 80 s and 120 s.

The control inputs of LQR and of the AVOECR-LQR observer-based control loop are shown in Figure 18 and Figure 19 for the scenario of Figure 14, respectively. Both controllers reject the fault between 80 s and 120 s. The control effort using the combined disturbance and fault observer is again slightly higher, because compensation terms were added to improve the rejection of disturbances, particularly their lower frequency contributions, and simultaneously to recover from the fault. The overall control effort is again very similar and clearly admissible.

The integrated control inputs over the simulation times of the climb cruise-land manoeuvre are given in Table 6 for the wind disturbance rejection comparison and in Table 7 for the wind and fault rejection comparison. It can be observed that even though the observer-based compensation naturally slightly increases overall integrated inputs (in absolute value), the increase is very moderate, from less than 1% for throttle to a maximum increase of 12.8% for the quad pitching moment, which is typically a practically feasible increase to achieve a much finer trajectory tracking. Using alternative observers, the added control effort was between the relatively close LQR and AVOECR-LQR values, but trajectory tracking was well below the performance obtained with AVOECR.

### 5.4. Discussion of Implementation Considerations and Extensions of the Approach

Implementation on a Pixhawk-4 based quadplane is part of the future work. The state estimates used as inputs to the perturbation observer were assumed not to be noisy, which is a common assumption in perturbation observers. States were estimated here using a Luenberger observer, but in practice, a Kalman filter is available open source within the PX4 toolchain for Pixhawk autopilots. Future work directions also include an extension of the approach to 6DoF flight control, adaptive observer-based controller tuning, as well as finite time convergence. The methods developed in this paper also extend to hybrid aircraft configurations and multimode systems more generally.

## 6. Conclusions

A quadplane model was developed and linearised for the longitudinal plane, quad and transition modes with speed based gradual transitioning. An unknown input observer based on the use of an auxiliary variable was then shown to accurately estimate either all three components of the wind gusts acting on the quadplane or the wind velocity components and an elevator fault. This observer was shown to significantly reduce estimation error compared to three classical observers based on output error integration, acceleration measurements and a sliding mode observer, respectively. The observer-based correction was also shown to substantially enhance the speed and altitude tracking performance of a linear quadratic regulator as well as a H-infinity controller, which were taken without loss of generality as benchmark controllers to be enhanced. Compared to the other observers, the integrated absolute error in altitude is reduced by a minimum of 30% with linear quadratic control and a minimum of 8% with H-infinity, which also reduces velocity error by 28%. The integrated absolute altitude error is further reduced by a factor of 3.41 to 4.55, compared to the use of benchmark controllers without an observer. The proposed approach is particularly efficient when the fault and wind disturbance are simultaneously active. Future work directions include an extension of this approach to six degrees of freedom dynamics and PX4 implementation.

## Figures and Tables

**Table 2:** Integrated absolute error for reference following with different perturbation observer gains and wind intensities.

Perturbation Observer Gain		IAE	
		Low Wind Intensity 2 m/s	High Wind Intensity 5 m/s
No observer correction	Velocity tracking	7.974	18.12
	Altitude tracking	162.6	266.2
*k* = 1	Velocity tracking	4.535	9.628
	Altitude tracking	126.7	173.2
*k* = 5	Velocity tracking	2.501	4.214
	Altitude tracking	113.8	125.1
*k* = 10	Velocity tracking	2.087	2.945
	Altitude tracking	110.9	117.3
*k* = 100	Velocity tracking	1.753	1.844
	Altitude tracking	108	110

**Table 3:** Integrated absolute error for wind velocities and fault estimation using all observers.

Estimated Disturbance Input	Observer	Comparison with Equivalent Observer Gains		Comparison When Alternative Observers Are Allowed Higher Gains	
		Observer Gain *k*	Disturbance Estimation IAE	Observer Gain *k*	Disturbance Estimation IAE
*u_g_*	AVOECR	100	2.352	100	2.352
	OEIO	100	66.55	500	12.12
	RAMO	100	108	500	34.47
	AVSMO	50	105.1	100	53.13
*w_g_*	AVOECR	100	9.198	100	9.198
	OEIO	100	83.07	500	77.49
	RAMO	100	128.7	500	53.1
	AVSMO	50	128.8	100	80.56
Elevator fault	AVOECR	100	0.3934	100	0.3934
	OEIO	100	0.8946	500	3.646
	RAMO	100	0.7922	500	2.2153
	AVSMO	50	1.4630	100	1.481

**Table 4:** IAE comparison of the observer-based disturbance rejection controllers based on LQR for the climb-cruise-land flight profile.

Controller	Altitude IAE	Velocity IAE
LQR	653.2	172
LQR with AVOECR	148.6	37.8
LQR with OEIO	193.6	49.93
LQR with RAMO	215.6	74.8
LQR with AVSMO	216.4	92.1

**Table 5:** Trajectory tracking IAE comparison of the observer-based disturbance rejection controllers based on H∞ control for the climb-cruise land flight profile.

Controller	Altitude IAE	Velocity IAE
H∞ controller	867.65	224.2
H∞ with AVOECR	254.81	56.6
H∞ with OEIO	272.64	72.28
H∞ with RAMO	332.4	115.9
H∞ with AVSMO	411.8	143.51

**Table 6:** Control effort comparison in the wind disturbance rejection case.

$\mathrm{Control}\;\mathrm{Input}\;u_{i}$, ith Element of u	Integrated Absolute Value of the Control Input $\int_{0}^{t_{f}}|u_{i}\left(t\right)|dt$	
	LQR	LQR with AVOECR Observer-Based Wind Disturbance Compensation
Plane elevator (*i* = 1)	915.7	959.8
Plane Throttle (*i* = 2)	5528	5530
Quad thrust (*i* = 3)	6326	6986
Quad pitching moment (*i* = 4)	1024	1156

**Table 7:** Control effort comparison in the wind and actuator fault rejection case.

$\mathrm{Control}\;\mathrm{Input}\;u_{i}$, ith Element of u	Integrated Absolute Value of the Control Input $\int_{0}^{t_{f}}|u_{i}\left(t\right)|dt$	
	LQR	LQR with AVOECR Observer-Based Disturbance and Fault Compensation
Plane elevator (*i* = 1)	922.8	963.1
Plane Throttle (*i* = 2)	5525	5530
Quad thrust (*i* = 3)	6337	7094
Quad pitching moment (*i* = 4)	1024	1161
